# Quality of Life and Relationships in Caregivers of People With Dementia. A Gender Perspective

**DOI:** 10.1177/15333175241276404

**Published:** 2024-08-19

**Authors:** Heidi Bjørge, Kari Kvaal, Ingun Ulstein

**Affiliations:** 1 60499 OsloMet – Oslo Metropolitan University, Faculty of Health, Institute of Nursing and Health Promotion, Oslo, Norway; 2 3207 Inland Norway University of Applied Sciences, Faculty of Health and Social Sciences, Elverum, Norway; 3 The Memory Clinic, Department of Geriatric Medicine, 155272 Oslo University Hospital Trust, Oslo, Norway

**Keywords:** dementia, caregivers, family, quality of life (QoL), gender, interpersonal relationship

## Abstract

Home-dwelling people with dementia rely on their family members to be able to stay at home. This affects the family caregivers’ quality of life (QoL). However, less is known about how male and female caregivers differ in their QoL. Our study aimed to investigate gender differences in caregivers’ quality of life (QoL), whether emotional relationships influence QoL, and how their QoL changes over time. The study applied a cross-sectional and a longitudinal design to examine a total of 208 caregivers, 158 female and 50 male caregivers, and their family member with dementia. Regression analysis and t-tests were performed to identify what characteristics about caregivers and care receivers influence male and female QoL, and whether caregivers’ QoL developed after one year. Both male and female caregivers’ depression influenced their QoL. For females, their own social distress influenced their QoL, and for males, their experience of their care receivers’ overemotional attitude influenced their QoL. From baseline to one-year follow-up their QoL decreased while their distress and experience of care receivers’ emotional attitudes was stable. Significant gender-specific differences were found, indicating that gender must be considered when approving caregivers’ needs and planning interventions for caregivers.

## Significance Statements

• Depressive symptoms were the strongest explanatory factor for QoL for both male and female caregivers.
• Female caregivers’ feelings of social distress contributed to reducing their QoL over time.
• Care receivers’ emotional over-involved attitude influenced on male caregivers’ QoL.

## Introduction

Most people with dementia live in the municipalities and depend on close relatives to be able to stay at home. Caring for a home-dwelling family member with dementia comes at a considerable cost for the caregiver, and impacts their quality of life (QoL).^1,2^ As dementia progresses, the cognitive decline, with memory loss, functional impairment and increased incidence of neuropsychiatric symptoms,^3^ comorbidities and mortality,^4,5^ imposes more responsibilities on the family caregiver.^6^ In addition, caregivers’ own health-related challenges including anxiety and depression may increase as well,^7^ and according to Lin et al,^8^ the role of caregiving hours mediates the burden. Although some describe their caregiving in positive terms and as rewarding, most studies underline the burden and stress that comes as the dementia progresses ^9-13^

An increasing number of studies has investigated what influences caregivers’ QoL. The most common factor associated with low caregiver QoL is caregiver depression.^1,14,15^ Other factors, such as the caregiver’s mental and physical health, general health, and sleep quality, have been shown to be related to QoL. Being a spousal caregiver is associated with poorer QoL in caregivers when compared with QoL in offspring caregivers.^1^

Gender is generally identified as an important contributor to the caregiving literature on the burden of care, as women and men may perceive their caregiving differently. However, studies have been contradictory in how male and female caregivers respond to the caregiver demands and thus how these demands influence their QoL. Most caregivers of persons with dementia are women.^16-18^ Female caregivers generally report significantly higher levels of burden and depression.^19-23^ Studies have described how women seem to provide more intense care for a longer period of time. They are more likely to support care for more basic needs and seem to find caregiving more physically and emotionally stressful.^21,24^ As opposed to females, male caregivers tend to be more task-oriented in their approach, and more reluctant to request municipal help.^20,25^ Male caregivers often feel their personal freedom diminish, and they struggle related to their gender identity when having to deal with new domestic tasks.^26,27^ Female caregivers often experience lower QoL,^28,29^ although there seems to be no conclusive results on whether male caregivers report higher levels of QoL.^1^

Relational factors have been discussed as a protective factor for QoL, but the literature on emotional relationships between caregiver and care receiver is sparse,^1^ and produces contradictory results. Studies have found that emotional closeness between caregiver and care receiver influence caregivers’ QoL.^30^ By contrast, a systematic review by Edwards et al.^31^ concludes that there is no evidence that relationship factors influence QoL in caregivers of people with dementia with respect to institutionalization, hospitalization or death, although a link between these factors has been extensively investigated.^31,32^ Feast et al.^33^ found that caregiver QoL was associated with the relationship quality and the caregiver’s sense of guilt, although with a weak association. In their systematic review and meta synthesis of qualitative research, Cross et al.^2^ found that the caregiver’s relationship with the care receiver was strained when the care receiver made accusations against the caregiver, which in turn affected the caregivers’ QoL. Using the concept of expressed emotion (EE) to assess relationship factors, Weisman de Mamani et al.^34^ found that caregivers’ perceived QoL was associated with attitudes of both criticism and emotional over-involvement. Caregivers’ sense of shame made them attempt to control care receivers’ negative behaviour related to the symptoms of dementia. This sense of shame influenced their QoL. EE refers to a specific psychological construct, and relates to a measure of the family environment based on how the relatives of a psychiatric patient spontaneously talk about or to the patient.

According to the stress process model,^35^ a good relationship is understood to modify the effect of the caregiver burden.^36^ Neuropsychiatric symptoms are the main contributors to caregiver burden and distress,^33,37^ hence a good relationship can modify the effect of intensified stress following an increase in dementia symptoms.^38^ However, there are few studies of how QoL in caregivers changes over time as the dementia progresses. Because dementia is a degenerative condition for which there is currently no cure, the role of caregiver will last for an extended period of time. Most of the studies on caregivers’ QoL describe no change in caregivers’ QoL despite a decline in the patient’s functioning over time.^38-41^ Riedijk et al.^42^ found that QoL was more affected in caregivers who had cared for their family member for a short period of time, and was more evident in younger caregivers. As Clare et al.^43^ underline, we know little about how QoL changes as dementia progresses or what factors influence such changes. Family caregivers of people with dementia are an important resource, thus it is important to ensure that their own QoL is satisfactory.^1^

Because a good QoL for caregivers might postpone their family member’ institutionalization,^31^ there is a need for more knowledge about what influences their ability to stay longer in their role as caregivers. Due to these inconclusive results, there is a need for more information about gender differences which could facilitate developing tailored care for men and woman.^44^

The overall aim was to investigate caregivers’ perceived QoL.

- Did caregivers’ QoL differ according to gender?
- Did caregivers’ perceived emotional relationship influence their QoL.
- Did caregivers’ perceived QoL change after one year?

## Methods

This study had both a cross-sectional and a longitudinal design. A total of 208 caregivers and their family member (dyads) with dementia took part in the study. They were recruited from memory clinics and home-based nursing services by local project co-workers in 19 municipalities in Norway. They were eligible to participate in the present study if the care receiver had been diagnosed with dementia according to ICD-10 criteria, scored at least 15 points on the Mini-Mental State Examination,^45^ and had the capacity to give informed written consent to participate in the study. The care receivers were living at home and had at least weekly face-to-face contact with the caregiver. No specific exclusion criteria were defined. The study used the baseline data from the enrolment and one-year follow-up data from a randomized controlled trial that aimed to examine the effect of a psychosocial intervention programme for home-dwelling persons with dementia and their caregivers.^46^ The psychosocial intervention consisted of education about dementia, counselling individually with each family, and group meetings where a problem-solving method was used to find new ways of coping with problems and unmet needs and lasted for 12 months. Of the original 230 participants from the randomized controlled trial,^46^ 22 participants were excluded from this study because they were more distant caregiver or friends, leaving only close relatives to achieve a more homogeneous material. The participants had attended either the intervention or the control group, and participants who remained in the study for one year were included in the last part of this study. During the follow-up period, 32 participants were lost due to declined health – leaving 176 dyads in the longitudinal part of the study. Eleven participants were lost due to the care receivers having died. Twenty-one participants withdrew for different reasons, such as the care receiver had moved to a nursing home, the caregiver had withdrawn, or were excluded for incomplete data.

The data were collected by trained nurses and occupational therapists in an interview session with the caregivers. Participants were enrolled from October 2009 to May 2011.

### Measures

The caregivers’ QoL was assessed using the Norwegian version of the 13-item Quality of Life-Alzheimer Disease questionnaire (QoL-AD),^47^ originally designed to rate QoL in patients. The questionnaire contains questions about family and friends, finances, physical and mental health and one general question about quality of life. Each item is rated on a four-point Likert scale, where 1 indicates poor QoL, 4 indicates excellent, with a total score between 13 and 52.^47^ A higher score indicates a better QoL. The questionnaire has been validated in a sample of people with dementia and showed good validity and reliability.^47^ The questionnaire has also been used to evaluate QoL in caregivers and has been validated in one similar sample with Cronbach’s alpha 0.86.^48^

Caregivers’ perceptions of their family member’s attitudes towards them were evaluated using the Felt Expresses Emotion Rating Scale (FEERS), which is a six-item questionnaire based on the EE theory. The items were divided into two factors: FEERS criticism containing questions regarding caregivers’ perceptions of being criticized, worried for or being controlled, and FEERS emotional over-involvement containing questions regarding caregivers’ perceptions of being treated as an independent person, being appreciated, and of being respected during the past month. The questions were rated on an anchored Likert scale from 0 (not at all) to 5 (to an extreme degree) where FEERS emotional over-involvement were inverted. FEERS has previously been used in a study of dementia with acceptable internal validity (Cronbach’s α: CC: 0.52, FEERS emotional over-involvement: 0.82 and Factor analysis FEERS criticism: 0.64–0.75 FEERS emotional over-involvement: 0.73–0.80.^49^

Depression in caregivers was measured using the Geriatric Depression Scale (GDS).^50^ The GDS consists of 30 items, scored as absent = 0 or present = 1. A total score of >11 indicates depression with a sensitivity of 84 % and a specificity of 95 %.^51^

The caregivers’ burden of care was completed using the Relatives’ Stress Scale (RSS),^52^ a questionnaire with 15 questions, scored at five levels of intensity, from 0 (not at all) to 4 (to a high degree). These scores were evaluated with a factor analysis method with a three-factor solution to identify emotional distress (6 items), social distress (e.g., feelings of being limited in terms of social life) (6 items), and negative feelings (3 items). The scale has been validated with good internal validity (Cronbach’s α: emotional distress: 0.84, social distress 0.86, and negative feelings 0.70).^53^

Neuropsychiatric symptoms in the care receivers were evaluated by proxy using the short form of the Neuropsychiatric Inventory (NPI-Q),^54^ which covers 12 items with a severity sum score ranging from 0 (no symptoms) to 36 (severe symptoms). In this study, we used a dichotomous scale to indicate the presence or absence of symptoms, and the total score ranged from 0 to 12.

Depression in the care receivers was assessed using the Cornell, a proxy-based questionnaire. It consisted of 19 items with a score of 0-2 for each item.^50^ A higher score indicated more symptoms of depression, with a total score between 0 and 38. A cut-off-score of ≥11 is considered to indicate probable cases of depression.^51^

The more complex instrumental activities that are required for independent living were shown using the Instrumental Activities of Daily Living Scale (I-ADL). The questionnaire includes functions such as cooking, shopping and managing finances.^55^ Each item was rated on a 3 to 5 level scale, from 1 (independent) to 5 (totally dependent), with a total score of 31.

Cognitive function in the care receiver was evaluated by using the Mini Mental State Examination (MMSE) by the interviewer.^45^ The scale consists of 20 items, rated on a scale from 0 to 30, where a higher score indicated better cognitive function.

Age, kinship, and living arrangements of both the caregiver and the care receiver were recorded along with the duration of symptoms and the type and amount of formal assistance.

The same questionnaires were completed at a one-year follow-up, and the results were compared with baseline scores.

### Statistical Analyses

Continuous variables were expressed as mean and standard deviation (SD). Categorical data were expressed as counts and proportions. The Spearman rank correlation coefficient was used to explore crude associations between the male and female caregivers’ QoL. Data from the three RSS variables (emotional distress, social distress and negative feelings) and the NPI variables were normally distributed. Thus, differences were tested with parametric tests. The FEERS criticism, GDS and Cornell data were skewed. Thus, differences were tested with non-parametric methods. For variables with 20% or fewer missing scores, the missing item scores were replaced with the total mean.

Unadjusted (bivariate) and adjusted (multiple) linear regression analyses were performed with the total QoL score divided by gender for comparison. Background characteristics of both the caregiver and the care receiver from Table 1 were included as independent variables and examined for significance and with unstandardized coefficient B. Variables with *P*-values of ≤.10 in the bivariate analyses were entered into the multivariate analysis. T-tests were performed to assess differences between variables from baseline to one-year follow-up. All tests were two-sided. *P*-values of ≤0.05 were considered statistically significant. Statistical analyses were performed with the statistical program SPSS version 23.

**Table 1.** Differences in Caregiver and Care Receiver Characteristics Baseline According to Caregivers’ Gender (N = 208).

	Female (n = 158)	Male (n = 50)	*P* Value
Spouses, n (%)	86 (54.4)	32 (64)	<0.001
Children, n (%)	72 (45.6)	18 (36)	<0.001
Caregiver age, mean (SD) range	62.5 (11.2) 35–87	67.9 (13.6) 40–89	0.02
Years of schooling, mean (SD)	12.5 (3.61)	13.0 (4.19)	0.40
Co-residing, n (%)	91 (57.6)	33 (66)	0.07
Daily contact, n (%)	95 (60.1)	36 (72)	0.36
Employment, n (%)	64 (40.5)	23 (46)	0.07
QoL, mean (SD) range	40.6 (5.4) 23–52	42.6 (5.0) 31–52	0.02
GDS, median (IQR) range	5.0 (2, 11) 0–27	3.5 (1, 7) 0–23	0.05
FEERS CC, median (IQR)	3.0 (1, 6) 0–14	2.5 (1, 5) 0–12	0.72
FEERS EOI, mean (SD)	6.4 (3.4) 0–15	4.9 (2.3) 0–10	0.001
RSS emotional distress, mean (SD) range	10.9 (4.8) 0–24	8.5 (5.5) 1–21	0.007
RSS social distress, mean (SD) range	10.3 (5.5) 0–24	9.1 (5.1) 0–22	0.20
RSS negative feeling, mean (SD) range	4.2 (2.1) 0–27	3.4 (2.3) 0–23	0.02
Care receiver age, mean (SD) range	78.5 (7.4) 58–94	78.4 (6.9) 60–88	0.97
Years of symptoms, mean (SD) range	4.3 (2.9) 0.2–14	4.5 (2.8) 1–11	0.66
MMSE, mean (SD) range	21.5 (3.6) 15–30	20.1 (3.3) 15–30	0.02
IADL, mean (SD) range	22.3 (6.8) 8–72	20.3 (6.2) 8–34	0.06
NPI, mean (SD) range	5.4 (2.5) 0–11	4.2 (2.6) 0–10	0.004
Cornell median (IQR)	7.0 (4, 11) 0–26	7.0 (3, 10) 0–28	0.54
Assistance, n (%)	107 (68)	40 (80)	0.19

IQR = interquartile range, QoL = Quality of life, range 13-52; FEERS CC = Felt Expressed Emotion Rating Scale; criticism, range 0-5. FEERS EOI = Felt Expressed Emotion Rating Scale; emotional over-involvement, range 0-5. RSS = Relatives’ Stress Scale, range 0-12; GDS = Geriatric Depression Scale, range 0-30; MMSE = Mini Mental State Examination, range 0-30; NPI = Neuropsychiatric Inventory (only presence of symptoms, range 0-12); IADL = Instrumental Activities of Daily Living, range 7-13. Cornell, range 0-38.

## Ethics Approval and Consent to Participate

The study followed the ethical principles outlined in the Declaration of Helsinki. The Regional Committee for Ethics in Medical Research, Southern-Eastern Norway and the Norwegian Data Inspectorate approved the study (NCTO 1287767). Both the person with dementia and the family caregiver received oral and written information about the study. Written informed consent was obtained from each participant. The participants were also informed that they could withdraw consent and discontinue participation in the study at any time without giving a reason. All participants were assured about the confidentiality of the gathered data.

## Results

Characteristics of the participants according to male and female caregivers’ measured baseline of 208 caregivers are shown in Table 1. Female caregivers accounted for the largest group of study participants, consisting of 86 spouses (age: 50-87, mean 71) and 72 daughters (age: 35-71, mean 53), while the group of male caregivers consisted of 32 husbands (age: 59-89, mean 77) and 18 sons (age: 40-63, mean 52). With a large number of daughters, female caregivers were younger than male caregivers and had a significantly lower QoL. They reported a higher degree of negative perceptions regarding their caregiving situation and their relationship with the care receiver. In addition, they cared for a person with a better cognitive function, but with a higher level of neuropsychiatric symptoms.

Regarding the children’s reporting from the male and female groups of caregivers separately, both daughters’ and sons’ QoL, their relationship with the care receiver, and their caring experiences were reported as being better than the female spouses and male spouses respectively. Both the daughters and the sons spent less time together with the care receiver. They cared for a person with the same level of cognitive function and neuropsychiatric symptoms as the male and female spouses, and their levels of distress were consistent with the spouses. The sons reported the same level of negative feelings as the male spouses.

### Bivariate Associations

By means of a linear regression analysis, we explored crude associations between selected variables. QoL was used as the dependent variable and was divided into male and female for comparison. Results from these analyses are shown in Table 2.

**Table 2.** Crude Associations Between QoL (Quality of Life) Divided Into Gender and Various Caregiver and Care Receiver Variables (N = 208).

Female	QoL			Male QoL		
	B	95% CI	*P*-value	B	95% CI	*P*-value
Caregiver age	−0.07	−0.14 – 0.007	0.08	−0.11	−0.22 – −0.01	0.03
Co-residing	−2.43	−4.10 – −0.75	0.005	−3.21	−6.09 – −0.33	0.03
Daily contact	1.21	0.33 – 2.08	0.007	1.46	−0.04 – 3.01	0.06
Employment	3.56	1.89 – 5.21	<0.001	3.08	0.14 – 6.02	0.04
GDS	−0.49	−0.60 – −0.39	<0.001	−0.59	−0.80 – −0.37	<0.001
FEERS CC	−0.50	−0.78 – −0.22	0.001	−0.80	−1.26 – −0.34	0.001
FEERS EOI	−0.42	−0.67 – 0.18	0.001	−1.10	−1.64 – −0.56	<0.001
RSS emotional distress	−0.43	−0.60 – 0.27	<0.001	−0.37	−0.61 – −0.13	0.003
RSS social distress	−0.46	−0.59 – −0.32	<0.001	−0.41	−0.67 – −0.16	0.002
RSS negative feeling	−0.57	−0.97 – −0.17	0.006	−0.99	−1.56 – −42	0.001
RSS total	−0.23	−0.31 – −0.16	<0.001	−0.20	−0.31– 0.09	0.001
Care receiver age	0.07	−0.05 – 0.18	0.26	0.06	−0.15 – 0.27	0.59
Care receiver gender	2.8	−4.50 – −1.15	0.001	2.0	−8.00 – −4.00	0.50
Years of symptoms	−0.19	−0.48 – −0.10	0.20	−0.15	−0.68 – 0.37	0.56
Years of schooling	−0.07	−0.31– 0.16	0.54	0.21	−0.41 – 0.82	0.50
MMSE	0.09	−0.15 – 0.33	0.45	0.57	0.16 – 0.98	0.008
IADL	−0.12	−0.25 – −0.001	0.05	−0.31	−0.53 – −0.19	0.006
NPI	−0.48	−0.82 – −0.15	0.005	0.01	−0.55 – −0.58	0.97
Cornell	−0.20	−0.35 – −0.05	0.011	−0.21	−0.45 – 0.04	0.09
Assistance	−0.19	−0.33 – 0.05	0.008	−0.32	−0.67 – −0.03	0.07

GDS = Geriatric Depression Scale, FEERS CC = Felt Expressed Emotion Rating Scale; criticism, FEERS EOI = Felt Expressed Emotion Rating Scale; emotional over-involvement, RSS = Relatives’ Stress Scale, MMSE = Mini Mental State Examination, IADL = Instrumental Activities of Daily Living, NPI = Neuropsychiatric Inventory.

Our data revealed that most of the caregivers’ own mood, distress and perceptions of their care receivers’ attitude and behaviour contributed to their reduced QoL. For the female caregivers, co-residence and the amount of contact, the care receivers’ gender and certain characteristics of their care receiver such as ADL function, mood and neuropsychiatric symptoms, contributed to their QoL. For the male caregivers, their own age, co-residing, and the characteristics of the care receivers, such as their cognitive function and ADL function, were associated with caregivers’ QoL.

There were no significant differences between the group of participants who participated or those who were excluded from this analysis (data not shown).

### Multivariate Analyses

Results from the multivariate analyses of caregiver and care receiver characteristics are listed in Table 3. The results show that caregivers’ own depressive feelings were the strongest independent predictor of both male and female caregivers’ QoL. Except for depressive symptoms, female caregivers’ RSS social distress predicted their QoL. Taken together, depressive symptoms and female caregivers’ RSS social distress accounted for 38% of the variance.

**Table 3.** Adjusted Linear Regression Analysis With the Dependent Variable QOL (Quality of Life) and Various Caregiver Variables According to Gender^a^ (N = 208).

Instrument	Female QoL			Male QoL		
	B	95% CI	*P*-value	B	95% CI	*P*-value
GDS	−0.45	−.52 – −.27	<0.001	−0.48	−.71 – −.25	<0.001
FEERS EOI				−0.54	−1.08 – −.01	0.05
RSS social distress	−0.21	−.36 – −.07	0.005			
*R*^2^ (%)	38			48		

^a^Only statistically significant explanatory variables (*P*-value ≤.05) are reported. RSS = Relatives’ Stress Scale; GDS = Geriatric Depression Scale; FEERS EOI = Felt Expressed Emotion Rating Scale; emotional over-involvement; RSS = Relatives’ Stress Scale.

For the male caregivers, both depressive symptoms and their perceived emotional over-involvement (FEERS emotional over-involvement) from the care receiver explained their QoL. Their depressive mood and emotional over-involvement accounted for 0.48% of the variance, but a large confidence interval between −1.08 and −0.01 of emotional over-involvement indicates a large variance in male caregivers’ perceptions of emotional over-involvement.

### Analyses of Variables Change Longitudinal

Table 4 shows the change in QoL and FEERS over time from inclusion to one-year follow-up by means of Spearmen rank correlation coefficient. As can be seen from the table, caregivers QoL decreased significantly over time, both for male and female caregivers, whereas other caregiver characteristics remained stable.

**Table 4.** Longitudinal Follow up by Means of Spearmen Rank Correlation Coefficient of Caregiver Change in Quality of Life (QoL) and Other Characteristics From Baseline to 12 months According to Gender (N = 176).

	Female			Male		
	Time 1	Time 2	*P* Value	Time 1	Time 2	*P* Value
QOL	40.9 (5.57)	23.9 (5.9)	<0.001	42.6 (5.1)	23.3 (5.2)	<0.001
FEERS EOI	6.34 (3.3)	6.58 (3.4)	0.41	5.0 (2.3)	5.76 (3.1)	0.11
GDS	7.08 (6.45)	7.23 (6.61)	0.70	5.45 (5.64)	5.62 (5.97)	0.79
RSS social distress	10.08 (5.67)	10.44 (6.01)	0.45	9.12 (5.39)	9.88 (6.35)	0.39

QoL = Quality of Life; FEERS EOI = Felt Expressed Emotion Rating Scale; emotional over-involvement; GDS = Geriatric Depression Scale; RSS = Relatives’ Stress Scale.

## Discussion

The main findings of the study show that caregivers’ depressive feelings were the strongest predictor of their reduced QoL. This was evident for both male and female caregivers, as in other studies.^1,2,14,30^ The female caregivers in our sample had higher levels of depressive feelings than male caregivers (mean score of 5 vs 3.5). These scores are evaluated as low when compared to other studies^20,44^ but still underline that even a low degree of depressive feelings has an impact on caregivers’ perceived QoL.

Further, the study revealed there were gender differences in what influenced caregivers’ QoL. Female caregivers’ feelings of social distress contributed to reducing their QoL which is consistent with previous findings.^1,12^ The studies by Farina et al.^1^ and Hu et al^56^ revealed that caregiver QoL was associated with caregivers’ own distress. However, our study goes further, as the association with distress is evident in female caregivers only. Their social distress might be seen in relation to giving care to a family member with higher levels of cognitive and neuropsychiatric symptoms than the male caregivers. Neuropsychiatric symptoms are related to caregiver burden in various studies. This objective burden might lead to caregivers having to spend more time neglecting their own needs for leisure time. Cross et al.^2^ found that caregivers who provided continuous care for the care receiver reported more distress due to not having time for themselves or their own family. They found themselves unable to continue the social roles they might have had prior to caregiving. Reflected in other studies, caregivers are in need for more time for themselves and for the possibility to continue their own interests.^1,57^

As opposed to female caregivers’ social distress, male caregivers’ QoL were influenced by their perception of care receivers’ emotional over-involved attitude towards them. In general, male caregivers experienced their caregiving role differently from female caregivers. They reported lower levels of depressive feelings and distress and higher levels of QoL. This fits well with Baker and Robertson,^27^ who describe the differences in male and female adaption to the caregiving role; they experience social isolation differently, and their caregiving role is experienced differently emotionally. Male caregivers’ QoL being affected by their care receivers’ emotional over-involved attitude towards them might be seen in relation to Clare et al.,^58^ who describe how cognitive deficits in people with dementia often result in a lack of awareness of their own disease as dementia progresses.^59^ This lack of awareness and increased neuropsychiatric symptoms may result in a negative attitude which is perceived as an emotional over-involved attitude. According to Cross et al.,^2^ care receivers’ negative attitude influenced the relationship between caregiver and care receiver and, consequently, caregivers’ QoL. However, the heterogeneous group of male caregivers consisting of both husbands and sons might result in differences in how they perceive their care receivers’ attitude. A large confidence interval in the adjusted linear regression analysis confirms the different attitudes and perceptions of their care receivers’ emotional over-involvement in them being a husband or being a son.

The follow-up data in our study reveal that caregivers’ QoL decreased over time, though more so for male than for female caregivers. The decrease in caregivers’ QoL is contrary to other studies, which report on the stability of caregivers QoL.^40-42^ In the study by Riedijk et al.,^60^ caregivers even described improvements in their QoL and in their psychological and physical well-being. The authors suggest that caregivers adapt to their caregiving demands over time. In line with this, Heru and Ryan^39^ found that caregivers experience more reward than burden. This differs from our results. This discrepancy might be due to the measures used to assess QoL. Several studies use different generic measures of QoL. Riedijk et al.^60^ used the Short Form 36 health survey questionnaire (SF-36) which is a generic health measure, while the QoL-AD is a measure specifically used to measure QoL in dementia. Although the questionnaire was designed to use on the patients, it may also capture the challenges caregivers meet better than a generic questionnaire does. Similar to other studies, different characteristics of caregivers remained stable in our study, such as caregivers’ depressive feelings and distress.

Our study revealed that male caregivers’ QoL was higher at baseline compared to female caregivers’ QoL, but that it decreased to the same level as the female caregivers at follow-up. Male caregivers’ QoL was influenced by their perceptions of their care receivers’ emotional over-involvement towards them. Emotional over-involvement is one of the components of EE which has shown that being exposed to a high degree of EE is associated with a worse prognosis in several mental illnesses. In dementia, Weisman de Mamani et al.^34^ found that when caregivers showed an attitude of both criticism and emotional over-involvement, this was associated with a decrease in caregivers’ QoL. To illustrate this further, the decrease in QoL in male caregivers over time in the present study can be seen in relation to studies on family members of people with schizophrenia where the concept of EE first occurred. In a study by Bentsen et al.,^61^ they found female caregivers to be more emotionally over-involved. In other EE studies, the level of EE is ascertained from the caregivers’ attitudes, whereas in our study we captured the caregivers’ perceptions of the care receivers’ attitudes and behaviour. Based on the knowledge of the negative outcome of high EE, male caregivers might seem to manage their caregiving role initially well, but over time their perception of their care receivers’ emotional over-involved attitude towards them causes their QoL to diminish.

Both the families and the health authorities share a common interest in caring for people in their own homes for as long as possible rather than in residential care. Appreciating the valuable resource caregivers represent for enabling care receivers to stay at home, the findings support taking gender considerations into account when planning for interventions, with special focus on the gender differences. Tailored interventions individually directed at their needs may lead to improvement in QoL.^62,63^ According to Walter and Pinquart,^64^ multicomponent psychoeducation and cognitive behavioural therapy interventions affect a broader range of outcomes. In addition, interventions should include some form of active participation or training. Particular attention should be paid to female caregivers’ feelings of social isolation in order to give them the possibility for leisure time, which has shown to improve their QoL.^1,65,66^ Several studies have found that cognitive-based interventions could have beneficial effects on caregivers’ QoL by reducing their depressive feelings, but no difference was found in caregiver relationship, functioning and burden.^67-69^ Given that dementia is a progressive, terminal disorder, caregiving is not a fixed set of experiences, and interventions should also address the changes in their caregiving career.^53,70^

The study revealed differences in male and female caregivers’ QoL which lead to the need for gender-specific multicomponent interventions. While female caregivers might need more time for leisure activities, male caregivers would need ways to deal with their perceived emotional over-involvement from their predominantly female care receivers. Information about dementia and the opportunity to discuss and address communication, contact, and closeness between the two might improve their relationship.

There are certain concerns related to the present study. The strength of the study is the small number of dropouts between recruitment and one-year follow-up. The care receivers were in a fragile stage of their life, and some did pass away during the follow-up. Still, most of the participants remained in the study. Further, we used a disease-specific measure of QoL instead of a generic questionnaire. The QoL-AD is a questionnaire designed to capture QoL in dementia patients. However, the questionnaire is also used in studies of caregivers^48,72,73^ and the questionnaire provides an easy way to rate several relevant issues for a caregiver of a person with dementia. The questionnaire measure several aspects of being a caregiver including the caregiving context, stressors, role strains, and resources, besides, the QoL-AD questionnaire benefit from a robust measure of Cronbach’s alpha.^48^ Generic questionnaires, as opposed to disease-specific measure of QoL, might fail to capture disease-specific elements crucial to QoL in the current condition and might not be sensitive enough to detect changes.^1,71^ Hence, several studies including measurement properties of the questionnaire would be appropriate for further use in research. The data came from a selected group of dyads recruited from memory clinics and home-based nursing services and thus not representative of the entire non-institutionalized population people with dementia and their caregivers. Male caregivers’ QoL was explained by the care receivers’ emotional over-involvement. However, a large intraclass correlation indicates diversity in the male caregivers’ perceptions, hence further research on male caregivers’ QoL is needed.

## Conclusions

Depressive symptoms were the strongest explanatory factor for QoL for both male and female caregivers. However, other significant gender-specific differences were found, indicating that gender must be considered in planning interventions for caregivers. As opposed to studies using generic measures of QoL, our study revealed that QoL decreased in caregivers. Further studies are needed to gain more knowledge in gender differences of QoL.

## Data Availability

The dataset used and analyzed during the current study are available from the corresponding author on request.*

## Appendix

### Abbreviations

QoL: Quality of life
EE: Expressed emotion
FEERS: Felt expressed emotion rating scale
CC: Critical comments
EOI: Emotional over-involvement
GDS: Geriatric depression Scale
MMSE: Mini mental state examination
RSS: Relatives’ stress scale
NPI-Q: Neuropsychiatric inventory
IADL: Instrumental activities of daily living
